# Liver and pancreas

**DOI:** 10.1186/1470-7330-15-S1-O24

**Published:** 2015-10-02

**Authors:** Jay P Heiken

**Affiliations:** 1 Mallinckrodt Institute of Radiology, Washington University School of Medicine, St. Louis, MO 63110, USA

## 

Various inflammatory processes and atypically appearing benign masses can have an imaging appearance very similar to or in some cases indistinguishable from cancer. In the liver such processes include inflammatory pseudotumor, sclerosed hemangioma and focal hepatic steatosis. In the pancreas heterotopic splenule, focal autoimmune pancreatitis and solid serous cystadenoma may be difficult to distinguish from pancreatic adenocarcinoma or neuroendocrine tumor.

*Inflammatory Pseudotumor* is a localized benign hepatic mass that consists of fibrous stroma and chronic inflammatory infiltrates including plasma cells, histiocytes and lymphocytes. Three subtypes are recognized: xanthogranuloma type (histiocyte predominance), plasma cell granuloma type and hyalinized sclerosing type. The pathogenesis is unclear, but it often is associated with chronic recurrent biliary infections. The most common CT/MR appearance of inflammatory pseudotumor is that of a peripherally enhancing mass with a central area of poor enhancement. Histopathologically, the peripheral enhancing portion of the mass corresponds to fibroblastic proliferation, whereas the central poorly enhancing area corresponds to chronic inflammatory infiltrates. Other appearances include a solid mass with progressive homogeneous enhancement (hyalinized sclerosing type) and a necrotic appearing mass with internal septations. Differential diagnosis includes abscess, metastasis, cholangiocarcinoma and hepatocellular carcinoma.

Hemangioma, the most common noncystic hepatic mass, has well recognized imaging characteristics; however, a hemangioma that has undergone degeneration and fibrous replacement (*sclerosed hemangioma*) may have atypical imaging features that can mimic a malignant hepatic neoplasm, in particular cholangiocarcinoma or metastasis. The imaging appearance varies depending on the degree of sclerosis. The mass often has an irregular contour and may cause capsular retraction if located peripherally. It may show rim enhancement or nodular peripheral enhancement; however, in contradistinction to most hemangiomas the enhancement is static and does show the usual centripetal progression. On MR imaging the mass may lack the expected degree of T2 hyperintensity. If serial imaging is available, a sclerosed hemangioma may decrease in size over time, with loss of previous regions of enhancement.

*Intrapancreatic splenule*appears as a round enhancing mass within the tail of the pancreas. Because of its vascularity it can be mistaken for a pancreatic neuroendocrine tumor. One of the keys to diagnosis is recognition that the mass has enhancement, attenuation and/or signal intensity characteristics that parallel the spleen on all image acquisitions. The diagnosis can be confirmed with a technetium 99m heat-damaged red blood cell scan, which demonstrates radiotracer uptake within the mass.

*Mass-forming chronic pancreatitis*, particularly *autoimmune pancreatitis*, frequently is misdiagnosed as pancreatic adenocarcinoma or a neuroendocrine tumor. Most commonly mass-forming chronic pancreatitis is isoattenuating/isointense during both the pancreatic and hepatic parenchymal phases of contrast enhancement. In contradistinction, pancreatic adenocarcinoma typically is hypoattenuating/hypointense during both enhancement phases; however, approximately 10% of pancreatic adenocarcinomas are isoattenuating/isointense during both phases. On MR cholangiopancreatography (MRCP) the pancreatic duct within the mass-forming pancreatitis may be visible but narrowed (duct-penetrating sign), whereas the duct within pancreatic carcinoma often is occluded. One study has shown the duct-penetrating sign to be 94% accurate in distinguishing the two entities. Elevation of serum IgG4 is the best serological marker for autoimmune pancreatitis (sensitivity 73-75%; specificity 93-95%); however, approximately 10% of patients with pancreatic cancer may have elevated IgG4.

Pancreatic *serous cystadenoma* is a benign mass that consists of numerous tiny cysts separated by glandular tissue and fibrous stroma. On CT it appears as a well-circumscribed hypoattenuating mass with varying degrees of contrast enhancement, depending on the size of the cysts and the proportion of cystic to glandular tissue. In most cases it is not difficult to distinguish this multicystic lesion from a solid pancreatic neoplasm; however a small proportion of serous cystadenomas consist largely of glandular tissue and fibrous septa with only a small proportion of tiny cysts. Such lesions appear hypervascular and may mimic a pancreatic neuroendocrine tumor. Clues to the diagnosis include precontrast attenuation value within the range of fluid and the presence of small cystic areas with the enhancing mass. In addition, this diagnosis should be considered if the mass is found incidentally in an elderly individual or a patient with von Hippel Lindau disease.
